# Internalized HIV stigma, ART initiation and HIV‐1 RNA suppression in South Africa: exploring avoidant coping as a longitudinal mediator

**DOI:** 10.1002/jia2.25198

**Published:** 2018-10-26

**Authors:** Valerie A Earnshaw, Laura M Bogart, Jean‐Philippe Laurenceau, Brian T Chan, Brendan G Maughan‐Brown, Janan J Dietrich, Ingrid Courtney, Gugulethu Tshabalala, Catherine Orrell, Glenda E Gray, David R Bangsberg, Ingrid T Katz

**Affiliations:** ^1^ Department of Human Development and Family Sciences University of Delaware Newark DE USA; ^2^ RAND Corporation Santa Monica CA USA; ^3^ Department of Psychological & Brain Sciences University of Delaware Newark DE USA; ^4^ Department of Medicine Brigham and Women's Hospital Boston MA USA; ^5^ Harvard Medical School Boston MA USA; ^6^ Southern Africa Labour and Development Research Unit University of Cape Town Rondebosch Cape Town South Africa; ^7^ Perinatal HIV Research Unit Faculty of Health Sciences University of the Witwatersrand Johannesburg South Africa; ^8^ Desmond Tutu HIV Centre, IDM and Department of Medicine University of Cape Town Cape Town South Africa; ^9^ South African Medical Research Council Cape Town South Africa; ^10^ Oregon Health & Science University‐Portland State University School of Public Health Portland OR USA; ^11^ Center for Global Health Massachusetts General Hospital Boston MA USA; ^12^ Harvard Global Health Institute Cambridge MA USA

**Keywords:** antiretroviral therapy, coping, HIV, South Africa, stigma

## Abstract

**Introduction:**

Cross‐sectional evidence suggests that internalized HIV stigma is associated with lower likelihoods of antiretroviral therapy (ART) initiation and HIV‐1 RNA suppression among people living with HIV (PLWH). This study examined these associations with longitudinal data spanning the first nine months following HIV diagnosis and explored whether avoidant coping mediates these associations.

**Methods:**

Longitudinal data were collected from 398 South African PLWH recruited from testing centres in 2014 to 2015. Self‐report data, including internalized stigma and avoidant coping (denying and distracting oneself from stressors), were collected one week and three months following HIV diagnosis. ART initiation at six months and HIV‐1 RNA at nine months were extracted from the South Africa National Health Laboratory Service database. Two path analyses were estimated, one testing associations between internalized stigma, avoidant coping and ART initiation, and the other testing associations between internalized stigma, avoidant coping and HIV‐1 RNA suppression.

**Results:**

Participants were 36 years old, on average, and 63% identified as female, 18% as Zulu and 65% as Xhosa. The two path models fit the data well (ART initiation outcome: *X* ^2^(7) = 8.14, *p *=* *0.32; root mean square error of approximation (RMSEA) = 0.02; comparative fit index (CFI) = 0.92; HIV‐1 RNA suppression outcome: *X* ^2^(7)  = 6.58, *p *=* *0.47; RMSEA = 0.00; CFI = 1.00). In both models, internalized stigma one week after diagnosis was associated with avoidant coping at three months, controlling for avoidant coping at one week. In turn, avoidant coping at three months was associated with lower likelihood of ART initiation at six months in the first model and lower likelihood of HIV‐1 RNA suppression at nine months in the second model. Significant indirect effects were observed between internalized stigma with ART non‐initiation and unsuppressed HIV‐1 RNA via the mediator of avoidant coping.

**Conclusions:**

Internalized stigma experienced soon after HIV diagnosis predicted lower likelihood of ART initiation and HIV‐1 RNA suppression over the first year following HIV diagnosis. Avoidant coping played a role in these associations, suggesting that PLWH who internalize stigma engage in greater avoidant coping, which in turn worsens medication‐ and health‐related outcomes. Interventions are needed to address internalized stigma and avoidant coping soon after HIV diagnosis to enhance treatment efforts during the first year after HIV diagnosis.

**Abbreviations**
ART: antiretroviral therapy
CD4: cluster of differentiation 4
NHLS: National Laboratory Service
PLWH: people living with HIV
RMSEA: root mean square error of approximation
UNAIDS: Joint United Nations Programme on HIV/AIDS

## 1 Introduction

The Joint United Nations Programme on HIV/AIDS’ (UNAIDS) goals for addressing the HIV epidemic include that 95% of people who have been diagnosed with HIV will have initiated antiretroviral therapy (ART) and 95% of people receiving ART will have suppressed HIV‐1 RNA by 2030 1. Initiating ART is a key step in the treatment cascade and necessary to achieve HIV‐1 RNA suppression, which in turn extends the lives of people living with HIV (PLWH) and prevents the transmission of HIV to others 2, 3, 4, 5. Reaching the goals set by UNAIDS may be difficult in places like South Africa, where only 61% of 7.2 million PLWH were receiving ART and 47% had suppressed HIV‐1 RNA in 2017 6. HIV stigma has long been recognized as a barrier to treatment and physical health among PLWH in South Africa and globally 7, 8, 9; yet, the mediating mechanisms linking HIV stigma with treatment and physical health outcomes remain understudied using prospective longitudinal designs 10. The current study addresses this gap by exploring longitudinal associations between HIV stigma, coping, ART initiation and HIV‐1 RNA suppression among PLWH in South Africa.

HIV stigma is conceptualized as a social process that exists when labelling, stereotyping, separation and discrimination of PLWH occur within a power structure 8. This social process, in turn, is experienced by individuals as a series of stigma mechanisms 11. Internalized stigma involves endorsing negative beliefs and feelings about PLWH and applying them to the self 11. Internalized stigma may be particularly high in the first few weeks after HIV diagnosis and then decreases over time 12. HIV stigma mechanisms are associated with lower likelihood of ART adherence 10, 13, and internalized HIV stigma appears to play a prominent role in treatment‐related behaviours. In a cross‐sectional study of PLWH in the United States, internalized stigma was associated with lower likelihood of ART adherence after controlling for enacted and anticipated stigma (i.e. past experiences of discrimination and future expectations of discrimination) 14. Other cross‐sectional studies in the United States and Canada have also found associations between internalized stigma and lower likelihood of ART adherence 15, 16 as well as ART initiation 17. Although generally understudied in longitudinal studies, one longitudinal study in the United States found no significant prospective association between internalized stigma and ART adherence one month later 18. No known longitudinal studies have examined the prospective association between internalized stigma and ART initiation.

Internalized stigma may be associated with a lower likelihood of ART initiation, in part, because it compromises adaptive coping. Coping is the process of managing demands created by stressful events that are perceived as exceeding one's resources 19, 20. Receiving an HIV diagnosis and needing to initiate ART may be experienced as stressful events 21 that exceed one's psychological, social and/or financial resources 22. Coping processes can be adaptive, helping people to respond to stressors in positive or healthy ways (e.g. approach or problem‐focused coping), or maladaptive, leading people to respond to stressors in negative or unhealthy ways (e.g. avoidant coping) 20. Internalized stigma may lead to maladaptive coping in part because it undermines hope and self‐esteem 23. Maladaptive coping, in turn, undermines engagement in health behaviours: Avoidant coping is associated with lower medication adherence and higher HIV‐1 RNA among PLWH 24.

We hypothesized that: (1) internalized stigma is associated with lower likelihoods of ART initiation and HIV‐1 RNA suppression over the first nine months post‐HIV diagnosis; and (2) maladaptive coping mediates these associations.

## 2 Methods

### 2.1 Context

The study took place between July 2014 and July 2015 in Soweto and Gugulethu, which are urban, densely populated townships in South Africa predominately inhabited by black South Africans. National estimates indicate a high prevalence of internalized stigma (43%) among PLWH in South Africa 9. In resource‐poor areas, internalized stigma may be particularly salient due to a strong interplay between socio‐economic inequalities and stigma, with higher socio‐economic status associated with lower internalized stigma 25. In an urban, resource‐poor area of Cape Town, 58% of patients referred for HIV treatment by a mobile clinic reported feeling either guilty of ashamed that they have HIV 26. Individuals were recruited from HIV testing centres for this study. At one site in Soweto, the testing centre was part of a research unit. Otherwise, testing centres were housed within community health centres wherein they were integrated with other care (e.g. primary care, HIV treatment).

### 2.2 Participants

Individuals were tested for HIV and immediately given their results. Those who tested positive had blood drawn for a CD4 count and were advised to return a week later for results. Participants included 500 ART‐eligible adults (18 years or older) who were recruited as they received their CD4 count results. ART eligibility was determined based on South African guidelines (CD4+ ≤ 350 cells/mm^3^ before 1 January 2015 and CD4+ < 500 cells/mm^3^ after 1 January 2015). Children and pregnant women were excluded from participating because they qualified for intensive adherence support.

### 2.3 Procedures

Trained multilingual interviewers, who were independent from the clinical staff, delivered an in‐person survey using Research Electronic Data Capture (REDCap) 27. Self‐report data, including internalized stigma and coping, were collected from participants one week following their HIV diagnosis and three months later. Medical data, including ART initiation and HIV‐1 RNA, were extracted from the National Health Laboratory Service (NHLS) database at six and nine months post‐baseline by a study team member. NHLS provides laboratory services to all public‐sector facilities in South Africa. Study procedures received Institutional Review Board approval from Human Subjects Committees at Partners Healthcare, the University of Witwatersrand Ethics Committee, the Gauteng Department of Health and the University of Cape Town Ethics Committee, and all participants provided informed consent.

### 2.4 Measures

Internalized HIV stigma was measured with the Internalized AIDS‐Related Stigma Scale 28. Other experiences of stigma, such as enacted and anticipated stigma, were not measured. The Internalized AIDS‐Related Stigma Scale asks participants whether they “agree” (1) or “disagree” (0) with six statements. Results of an exploratory factor analysis using the baseline data suggested a two‐factor solution accounting for 69.25% of the variance in the scale. The first factor included four internalized stigma items capturing feelings of shame, worthlessness, guilt and dirty, and the second factor included two disclosure concerns items. The disclosure items were therefore dropped from the scale. The remaining internalized stigma subscale had adequate reliability (Cronbach's alpha = 0.81), and a mean composite score was created to represent internalized stigma.

Maladaptive coping was measured with 12 items from the Brief COPE measuring self‐distraction, denial, substance use, disengagement, venting and self‐blame 29. Items from the self‐blame subscale regarding self‐criticism and self‐blame that conceptually overlapped with internalized stigma were first removed. An initial factor analysis suggested a three‐factor solution accounting for 62.34% of the variance in the scale; however, it included one item that did not load well onto any subscale (*“*I've been saying to myself ‘this isn't real*’”*; factor loadings < 0.40). After this item was deleted, an additional factor analysis was conducted that suggested a two‐factor solution accounting for 54.50% of the variance in the scale. One factor included six items related to avoidance, denial and negative emotion release. This avoidance subscale had adequate reliability (Cronbach's alpha = 0.76). A mean composite score was created to represent the construct.

Following previously validated procedures for imputing dates of treatment initiation using NHLS data 30, ART initiation by six months was determined based on a measure of creatinine. Plasma RNA results were directly entered into NHLS by clinical providers, and HIV‐1 RNA suppression by nine months was calculated (<50 copies/mL).

Participants reported their age, sex, ethnicity, relationship status, education and employment at the baseline study interview. They also reported their binge drinking (calculated from number of drinks per day), recreational drug use and tobacco use in the past 30 days.

### 2.5 Analyses

First, descriptive analyses were conducted using SPSS 31 to characterize the socio‐demographic and other relevant characteristics of the sample. Second, all correlations between internalized stigma at one week after HIV diagnosis, avoidant coping at three months, ART initiation at six months and HIV‐1 RNA suppression at nine months were examined. Correlations between socio‐demographic characteristics and these variables were also explored. Pearson's correlations were used for correlations between continuous variables, point‐biserial between continuous and dichotomous variables, and Spearman's rho between dichotomous variables.

Third, two path analyses were conducted using Mplus version 8 32 to test for longitudinal mediation 33. The first model included ART initiation as the dependent variable, and the second model included HIV‐1 RNA suppression as the dependent variable. Associations between internalized stigma at one week post‐diagnosis with avoidant coping at three months post‐diagnosis were modelled following recommendations for longitudinal analyses by controlling for avoidant coping at one week 34. Associations between avoidant coping at three months with ART initiation at six months post‐diagnosis and HIV‐1 RNA suppression at nine months post‐diagnosis were also modelled. The effects of socio‐demographic characteristics and substance use that were correlated with the mediator (avoidant coping) and/or outcomes (ART initiation and HIV‐1 RNA suppression) were controlled for on both the mediator and outcomes, and models controlled for clustering by study site via standard error adjustment using a sandwich estimator. Socio‐demographic characteristics and substance use variables that were not statistically significant in the multivariate path analyses were trimmed from the final model 35. Following recommendations for mediation testing with dichotomous outcomes 36, 37, 38, counterfactually based indirect effects for links between baseline internalized HIV stigma and the distal outcomes ART initiation and HIV‐1 RNA suppression via the mediator of three‐month avoidant coping were estimated.

## 3 Results

Of the 500 participants enrolled in the study, 14 (2.8%) did not respond to one of the substance use questions at baseline and an additional 88 (18.5%) did not complete the three‐month survey. These 102 individuals with missing data were excluded from the analyses, resulting in an analytic sample of 398 participants. Sample characteristics, including socio‐demographic data, are displayed in Table 1 and reflected those of patients at the data collection sites. Participants who did not identify as Zulu or Xhosa identified as Sesotho (5.8%), Tsonga (2.3%) or another ethnicity (e.g. Tswanga, Venda, N. Sotho). Fewer participants who were single (17.6%; *X* ^*2*^(1) = 5.69, *p *=* *0.02) or virally suppressed (14.7%; *X* ^*2*^(1) = 7.24; *p *=* *0.01) were excluded from the analytic sample than those who were included. Conversely, more engaged in recreational drug use (18.6%; *X* ^*2*^(1) = 36.23, *p *<* *0.01).

**Table 1:** Sample characteristics (n = 398)

	Mean (SD)	% (n)
Socio‐demographic characteristics		
Age	36.11 (9.24)	
Female		63.3 (252)
Zulu ethnicity		17.6 (70)
Xhosa ethnicity		64.6 (257)
Single relationship status		70.6 (281)
High school education		26.1 (104)
Employed		43.5 (173)
Recreational drug use		2.8 (11)
Binge drinking		46.2 (184)
Tobacco use		24.4 (97)
Internalized stigma, 1 week	0.26 (0.34)	46.5 (185)
Avoidant coping		
1 week	1.60 (0.68)	
3 months	1.32 (0.55)	
ART initiation, 6 months		57.3 (228)
HIV‐1 RNA suppression, 9 months		27.6 (110)

Internalized stigma scores ranged 0 to 1 and coping scores ranged 1 to 4. The mean internalized stigma score and percentage of participants who agreed with at least one internalized stigma item are reported.

ART, antiretroviral therapy.

As shown in Table 2, internalized stigma at one week after HIV diagnosis was positively correlated with avoidant coping at three months. Avoidant coping was negatively correlated with ART initiation at six months and HIV‐1 RNA suppression at nine months.

**Table 2:** Longitudinal correlations

	Internalized stigma, 1 week	Avoidant coping, 3 months	ART initiation, 6 months	HIV‐1 RNA suppression, 9 months
Avoidant coping, 3 months	0.23**	1		
ART initiation, 6 months	−0.03	−0.10*	1	
HIV‐1 RNA suppression, 9 months	0.02	−0.13**	0.53**	1
Age	−0.05	−0.01	0.08	0.04
Female	−0.01	−0.01	0.05	0.04
Zulu ethnicity	0.07	0.33**	−0.11*	−0.09
Xhosa ethnicity	−0.22**	−0.48**	0.14**	0.13*
Single relationship status	−0.01	−0.01	−0.06	−0.10
High school education	−0.05	−0.20**	0.04	0.05
Employed	0.01	−0.06	−0.01	0.01
Recreational drug use	0.11*	0.17**	−0.04	−0.04
Binge drinking	−0.02	−0.01	−0.01	−0.03
Tobacco use	−0.06	−0.05	−0.04	−0.01

**p <* *0.01, *p <* *0.05.

ART, antiretroviral therapy.

**Figure 1:**
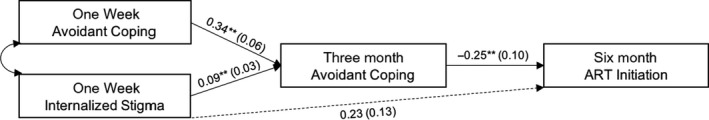
Longitudinal structural equation model of associations between internalized stigma, avoidant coping and antiretroviral therapy initiation. Values are unstandardized regression coefficients. Model controls for socio‐demographics and substance use on avoidant coping at three months and ART initiation at six months. **p < 0.01

The first model, predicting ART initiation at 6 months, demonstrated good fit to the data (*X* ^2^(7) = 8.14, *p *=* *0.32; RMSEA = 0.02 (90% CI = 0.00 to 0.07); comparative fit index (CFI) = 0.92). As shown in Figure 1, internalized stigma at one week was associated with avoidant coping at three months. Avoidant coping at three months was associated with lower likelihood of ART initiation at six months. Additionally, participants who were not Xhosa (*B*(SE) = −0.31(0.13), *p *=* *0.01) engaged in more avoidant coping. The remaining socio‐demographic variables and substance use variables were not associated with avoidant coping or ART initiation. A significant counterfactually based indirect effect of internalized stigma on ART initiation via avoidant coping was also observed (total natural indirect effect: OR = 1.04, *p *=* *0.04).

**Figure 2:**
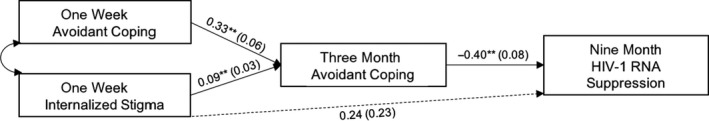
Longitudinal structural equation model of associations between internalized stigma, avoidant coping and HIV‐1 RNA suppression. Values are unstandardized regression coefficients. Model controls for socio‐demographics and substance use on avoidant coping at three months and HIV‐1 RNA suppression and nine months. **p < 0.01

The second model, predicting HIV‐1 RNA suppression at 9 months, also demonstrated good fit to the data (*X* ^2^(7)  = 6.58, *p *=* *0.47; RMSEA = 0.00 (90% CI = 0.00 to 0.06); CFI = 1.00). As shown in Figure 2, internalized stigma at one week was again associated with avoidant coping at three months. Avoidant coping at three months was associated with lower likelihood of HIV‐1 RNA suppression at nine months. Additionally, participants who were not Xhosa, engaged in more avoidant coping (*B*(SE) = −0.34(0.13), *p *=* *0.01). The remaining socio‐demographic variables and substance use were not associated with avoidant coping or HIV‐1 RNA suppression in this model. A significant counterfactually based indirect effect of internalized stigma on HIV‐1 RNA suppression via avoidant coping was also observed (total natural indirect effect: OR = 1.07, *p *<* *0.01).

## 4 Discussion

Results of this longitudinal study of 398 South African PLWH contribute to understanding of whether and how internalized HIV stigma is associated with deleterious HIV treatment and physical health outcomes. Previous cross‐sectional work has suggested that internalized HIV stigma is associated with a lower likelihood of ART initiation 17. The current study extends this research by demonstrating that internalized stigma measured one week after HIV diagnosis is prospectively associated with both non‐initiation of ART at six months as well as unsuppressed HIV‐1 RNA at nine months. The associations between internalized stigma and these outcomes were mediated, and results reflected inconsistent mediation (i.e. the signs of the mediated effect were opposite of the signs of the direct effects) 38. In cases of inconsistent mediation, the direct effect between an independent variable and dependent variable is non‐significant despite the existence of a significant indirect effect between the variables 38. In the current study, the direct effects between internalized stigma with ART initiation and HIV‐1 RNA suppression were non‐significant despite the existence of significant indirect effects between these variables. One previous longitudinal study did not find a direct association between internalized stigma and ART adherence 18. This non‐significant finding may have also been due to inconsistent mediation (i.e. internalized stigma may have been associated with ART adherence in this previous study via an untested mediator) or differences in the dependent variables (i.e. internalized stigma may play different roles in ART initiation vs. adherence).

Our results further suggest that maladaptive coping mediates associations between internalized HIV stigma with ART initiation and HIV‐1 RNA suppression. Internalized HIV stigma may lead PLWH to engage in efforts to avoid, deny and distract themselves from stressors, which in turn reduces the likelihood of ART initiation and HIV‐1 RNA suppression. Previous cross‐sectional studies have identified depressive symptoms, loneliness, low perceived social support, attachment‐related anxiety and HIV disclosure concerns as mediators of associations between internalized HIV stigma and ART‐related outcomes, including initiation and adherence 15, 16, 17. It is possible that the process linking internalized HIV stigma with ART initiation and HIV‐1 RNA involves serial mediation of these psychosocial variables. For example, internalized HIV stigma may lead to greater depressive symptoms, which in turn may lead to greater avoidant coping, ART non‐initiation and unsuppressed HIV‐1 RNA. The process linking internalized HIV stigma with ART non‐initiation and HIV‐1 RNA non‐suppression may also involve multiple pathways. Future research exploring multiple psychosocial mediators simultaneously can contribute to stronger understanding of the mechanisms linking internalized HIV stigma with HIV treatment and health outcomes.

### 4.1 Strengths, limitations and future directions

These results add to the literature on associations between internalized HIV stigma with treatment and physical health outcomes. Much of the previous research examining these associations has been cross‐sectional 14, 15, 17, limiting possibilities for forming firm conclusions regarding prospective associations between variables. The current study answers a call for longitudinal research testing prospective associations between stigma and ART outcomes, and for identifying mediators of these associations 10. Results of this longitudinal study suggest that internalized HIV stigma one week after HIV diagnosis is associated with avoidant coping at three months, controlling for avoidant coping one week after HIV diagnosis. Moreover, results suggest that internalized stigma one week after HIV diagnosis is associated with lower likelihoods of ART initiation at six months and HIV‐1 RNA suppression at nine months. Direct effects between internalized stigma with ART initiation and HIV‐1 RNA suppression were not statistically significant, but indirect effects reflecting inconsistent mediation were observed. Future researchers examining associations between internalized stigma with similar outcomes should note that indirect effects may exist between these variables even if direct effects are not statistically significant, and therefore, it may be important to explore mediators to fully understand these associations.

The current study focused on the role of internalized HIV stigma in treatment and physical health outcomes. Other studies have focused on other HIV stigma mechanisms, including enacted and/or anticipated stigma 13, 39. Future research that includes multiple HIV stigma mechanisms can contribute to our understanding of which stigma mechanisms are the most robust predictors of HIV treatment and health outcomes. Additionally, participants may experience stigma associated with other socially devalued characteristics (e.g. race, gender). Future longitudinal research should examine the extent to which and mechanisms whereby these other forms of stigma are associated with HIV treatment and physical health outcomes. Future research may also include measures of resilience resources, such as adaptive coping strategies and social support, to provide insight into who is resilient to the deleterious effects of HIV stigma.

ART initiation by six months after HIV diagnosis was abstracted from South Africa's NHLS. Participants may have initiated ART before six months. Prior research focused on associations between ART initiation and subsequent changes in internalized HIV stigma 40 suggest that ART initiation may lead to decreases in internalized HIV stigma. Future studies incorporating more precise data on when ART is initiated can continue to clarify temporal associations between stigma, coping and ART initiation. Future work should measure ART adherence in addition to ART initiation and HIV‐1 RNA to further elucidate associations between internalized stigma, coping and these outcomes. Participants who provided incomplete data were excluded from the analyses. Fewer single and virally suppressed participants provided incomplete data than those who were included in the analyses, and more engaged in recreational drug use. Future research is needed to examine the extent to which findings generalize to participants who were underrepresented in the current analysis. Finally, it is important to study these associations within South Africa given findings suggesting that over 40% of the 7.2 million PLWH living in South Africa have internalized some degree of stigma, 39% are not currently receiving ART and 53% have unsuppressed HIV‐1 RNA 6, 9. Yet, future research is needed to determine whether these findings generalize to other sociocultural contexts.

## 5 Conclusions

Greater understanding of the role of internalized stigma in HIV treatment and physical health outcomes, including the mechanisms linking stigma with these outcomes, can inform intervention efforts to promote the wellbeing of PLWH. The current study suggests that it may be important to address internalized HIV stigma and maladaptive coping among individual PLWH to promote ART initiation and HIV‐1 RNA suppression. There is some evidence to suggest that livelihood interventions that enhance economic empowerment may reduce internalized HIV stigma, possibly by weakening symbolic associations between HIV and economic incapacity, illness and death 41, 42. Interventions that focus on other forms of empowerment, such as pride, may further reduce internalized stigma 43. It may also be possible to teach or strengthen adaptive coping strategies (e.g. support seeking) and decrease use of maladaptive coping strategies (e.g. avoidance and denial). Interventions involving coping effectiveness training and teaching cognitive behavioural stress management techniques have led to improved psychosocial outcomes, including reduced stress, among PLWH 20, 44. It may be particularly important to assess and address internalized stigma and coping strategies soon after HIV diagnosis to facilitate timely initiation of ART. Ultimately, it is important to eliminate HIV stigma so that no individual person diagnosed with HIV is at risk of internalizing HIV stigma and experiencing the negative downstream effects of internalized HIV stigma on diminished coping, ART non‐initiation and unsuppressed HIV‐1 RNA. Researchers, clinicians and policymakers should continue to develop and evaluate strategies to address HIV stigma and promote the wellbeing of PLWH in South Africa and globally.

## Competing Interests

The authors have no competing interests.

## Authors’ Contributions

VAE conceptualized, conducted and interpreted the data analyses with contributions from LMB, JPL, BTC, BGMB and ITK. VAE wrote the first draft of the manuscript. ITK designed and led the study with guidance and oversight from LMB, JJD, IC, GT, CO, GEG and DRB. All authors assisted with the writing and revising of the manuscript, provided final approval of the version to be published and agreed to be accountable for all aspects of the work.

## References

[1] The Joint United Nations Programme on HIV/AIDS (UNAIDS) . Fast‐track: Ending the AIDS epidemic by 2030. Geneva, Switzerland; 2014. [cited: 2018 Oct 17] Available from: http://www.unaids.org/sites/default/files/media_asset/JC2686_WAD2014report_en.pdf

[2] World Health Organization . Guideline on when to start antiretroviral therapy and on pre‐exposure prophylaxis for HIV. Geneva, Switzerland: WHO Press; 2015. ISBN: 978‐92‐4‐150956‐5. 26598776

[3] Cohen MS , Chen YQ , McCauley M , Gamble T , Hosseinipour MC , Kumarasamy N , et al. Prevention of HIV‐1 infection with early antiretroviral therapy. N Engl J Med. 2011;365(6):493–505. 21767103 10.1056/NEJMoa1105243 PMC3200068

[4] The INSIGHT START Study Group . Initiation of antiretroviral therapy in early asymptomatic HIV infection. N Engl J Med. 2015;2015(373):795–807. 10.1056/NEJMoa1506816 PMC4569751 26192873

[5] The TEMPRANO ANRS 12136 Study Group . A trial of early antiretrovirals and isoniazid preventive therapy in Africa. N Engl J Med. 2015;2015(373):808–22. 10.1056/NEJMoa1507198 26193126

[6] UNAIDS . Overview. South Africa [Internet]. 2018 [cited: 2018 Oct 17]. Available from: http://www.unaids.org/en/regionscountries/countries/southafrica

[7] Aggleton P , Parker RA . A conceptual framework and basis for action: HIV/AIDS stigma and discrimination. Geneva, Switzerland: Joint United Nations Programme on HIV/AIDS; 2002.

[8] Mahajan AP , Sayles JN , Patel VA , Remien RH , Ortiz D , Szekeres G , et al. Stigma in the HIV/AIDS epidemic: a review of the literature and recommendations for the way forward. AIDS. 2008;22 Suppl 2:S67. 10.1097/01.aids.0000327438.13291.62 PMC2835402 18641472

[9] Leickness S , Khangelani Z , Allanise C , Sean J , Siphiwo Z , Sindisiwe B , et al. The people living with HIV stigma index: South Africa 2014: summary report. Pretoria, South Africa: Human Sciences Research Council; 2015.

[10] Sweeney SM , Vanable PA . The association of HIV‐related stigma to HIV medication adherence: a systematic review and synthesis of the literature. AIDS Behav. 2016;20(1):29–50. 26303196 10.1007/s10461-015-1164-1

[11] Earnshaw VA , Chaudoir SR . From conceptualizing to measuring HIV stigma: a review of HIV stigma mechanism measures. AIDS Behav. 2009;13:1160–77. 19636699 10.1007/s10461-009-9593-3 PMC4511707

[12] Eaton LA , Earnshaw VA , Maksut JL , Thorson KR , Watson RJ , Bauermeister JA . Experiences of stigma and health care engagement among Black MSM newly diagnosed with HIV/STI. J Behav Med. 2018;41:458–66. 29626312 10.1007/s10865-018-9922-y PMC6031458

[13] Katz IT , Ryu AE , Onuegbu AG , Psaros C , Weiser SD , Bangsberg DR , et al. Impact of HIV‐related stigma on treatment adherence: systematic review and meta‐synthesis. J Int AIDS Soc. 2013;16:18640. 24242258 10.7448/IAS.16.3.18640 PMC3833107

[14] Earnshaw VA , Smith LR , Chaudoir SR , Amico KR , Copenhaver MM . HIV stigma mechanisms and well‐being among PLWH: a test of the HIV Stigma Framework. AIDS Behav. 2013;17(5):1785–95. 23456594 10.1007/s10461-013-0437-9 PMC3664141

[15] Turan B , Smith W , Cohen MH , Wilson TE , Adimora AA , Merenstein D , et al. Mechanisms for the negative effects of internalized HIV‐related stigma on antiretroviral therapy adherence in women: the mediating roles of social isolation and depression. J Acquir Immune Defic Syndr. 2016;72(2):198. 26885803 10.1097/QAI.0000000000000948 PMC4868649

[16] Helms CB , Turan JM , Atkins G , Kempf M‐C , Clay OJ , Raper JL , et al. Interpersonal mechanisms contributing to the association between HIV‐related internalized stigma and medication adherence. AIDS Behav. 2016;21:238–47. 10.1007/s10461-016-1320-2 PMC4980279 26864692

[17] Logie CH , Lacombe‐Duncan A , Wang Y , Kaida A , Conway T , Webster K , et al. Pathways from HIV‐related stigma to antiretroviral therapy measures in the HIV care cascade for women living with HIV in Canada. J Acquir Immune Defic Syndr. 2018;77(2):144–53. 29135650 10.1097/QAI.0000000000001589 PMC5770113

[18] Kalichman SC , Grebler T . Stress and poverty predictors of treatment adherence among people with low‐literacy living with HIV/AIDS. Psychosom Med. 2010;72(8):810–6. 20716711 10.1097/PSY.0b013e3181f01be3 PMC3016469

[19] Lazarus RS , Folkman S . Stress, appraisal, and coping. New York: Springer; 1984.

[20] Taylor SE , Stanton AL . Coping resources, coping processes, and mental health. Annu Rev Clin Psychol. 2007;3:377–401. 17716061 10.1146/annurev.clinpsy.3.022806.091520

[21] Nightingale VR , Sher TG , Hansen NB . The impact of receiving an HIV diagnosis and cognitive processing on psychological distress and posttraumatic growth. J Trauma Stress. 2010;23(4):452–60. 20648562 10.1002/jts.20554 PMC3629914

[22] Thompson SC , Nanni C , Levine A . The stressors and stress of being HIV‐positive. AIDS Care. 1996;8(1):5–14. 8664369 10.1080/09540129650125957

[23] Yanos PT , Roe D , Markus K , Lysaker PH . Pathways between internalized stigma and outcomes related to recovery in schizophrenia spectrum disorders. Psychiatr Serv. 2008;59(12):1437–42. 19033171 10.1176/appi.ps.59.12.1437 PMC2605316

[24] Weaver KE , Llabre MM , Durán RE , Antoni MH , Ironson G , Penedo FJ , et al. A stress and coping model of medication adherence and viral load in HIV‐positive men and women on Highly Active Antiretroviral Therapy (HAART). Health Psychol. 2005;24:385–92. 16045374 10.1037/0278-6133.24.4.385

[25] Tsai AC . Socioeconomic gradients in internalized stigma among persons with HIV in sub‐Saharan Africa. AIDS Behav. 2015;19(2):270–82. 25572833 10.1007/s10461-014-0993-7 PMC4344381

[26] Maughan‐Brown B , Smith P , Kuo C , Harrison A , Lurie MN , Bekker LG , et al. Readiness for antiretroviral therapy: implications for linking HIV‐infected individuals to care and treatment. AIDS Behav. 2018;22(3):691–700. 28752353 10.1007/s10461-017-1834-2 PMC5785568

[27] Harris PA , Taylor R , Thielke R , Payne J , Gonzalez N , Conde JG . Research electronic data capture (REDCap) – a metadata‐driven methodology and workflow process for providing translational research informatics support. J Biomed Inform. 2009;42(2):377–81. 18929686 10.1016/j.jbi.2008.08.010 PMC2700030

[28] Kalichman SC , Simbayi LC , Cloete A , Mthembu PP , Mkhonta RN , Ginindza T . Measuring AIDS stigmas in people living with HIV/AIDS: the Internalized AIDS‐Related Stigma Scale. AIDS Care. 2009;21:87–93. 19085224 10.1080/09540120802032627

[29] Carver CS . You want to measure coping but your protocol's too long: consider the brief COPE. Int J Behav Med. 1997;4:92–100. 16250744 10.1207/s15327558ijbm0401_6

[30] Maskew M , Bor J , Hendrickson C , MacLeod W , Bärnighausen T , Pillay D , et al. Imputing HIV treatment start dates from routine laboratory data in South Africa: a validation study. BMC Health Serv Res. 2017;17(1):41. 28095905 10.1186/s12913-016-1940-2 PMC5240407

[31] IBM Corp . IBM SPSS statistics for Windows. Armonk, NY: IBM Corp.

[32] Muthén LK , Muthén BO . Mplus version 8. Los Angeles, CA: Muthén & Muthén; 2017.

[33] Muthén LK , Muthén BO . Mplus User's Guide. 8th ed. Los Angeles, CA: Muthén & Muthén; 1998‐2017.

[34] Maxwell SE , Cole DA . Bias in cross‐sectional analyses of longitudinal mediation. Psychol Methods. 2007;12(1):23. 17402810 10.1037/1082-989X.12.1.23

[35] Kline R . Principles and practice of structural equation modeling, 3rd ed. New York: Guilford Press; 2011.

[36] Muthén B , Asparouhov T . Causal effects in mediation modeling: an introduction with applications to latent variables. Struct Equ Modeling. 2015;22(1):12–23.

[37] VanderWeele TJ . Explanation in causal inference: methods for mediation and interaction. New York: Oxford University Press; 2015.

[38] MacKinnon DP , Fairchild AJ . Current directions in mediation analysis. Curr Dir Psychol Sci. 2009;18:16–20. 20157637 10.1111/j.1467-8721.2009.01598.x PMC2821103

[39] Bogart LM , Landrine H , Galvan FH , Wagner GJ , Klein DJ . Perceived discrimination and physical health among HIV‐positive Black and Latino men who have sex with men. AIDS Behav. 2013;17:1431–41. 23297084 10.1007/s10461-012-0397-5 PMC3631464

[40] Tsai AC , Bangsberg DR , Mwebesa B , Haberer JE , Frongillo EA , Muzoora C , et al. How does antiretroviral treatment attenuate the stigma of HIV? Evidence from a cohort study in rural Uganda. AIDS Behav. 2013;17:2725–31. 23670710 10.1007/s10461-013-0503-3 PMC3773278

[41] Tsai AC , Abigail Hatcher BM , Elizabeth Bukusi BA , Weke E , Lee Lemus Hufstedler B , Shari Dworkin BL , et al. A livelihood intervention to reduce the stigma of HIV in rural Kenya: longitudinal qualitative study. AIDS Behav. 2017;21:248–60. 26767535 10.1007/s10461-015-1285-6 PMC5444205

[42] Maluccio JA , Wu F , Rokon RB , Rawat R , Kadiyala S . Assessing the impact of food assistance on stigma among people living with HIV in Uganda using the HIV/AIDS Stigma Instrument‐PLWA (HASI‐P). AIDS Behav. 2017;21(3):766–82. 27372803 10.1007/s10461-016-1476-9

[43] Earnshaw VA , Bogart LM , Dovidio JF , Williams DR . Stigma and racial/ethnic HIV disparities: moving toward resilience. Am Psychol. 2013;68(4):225–36. 23688090 10.1037/a0032705 PMC3740715

[44] Bogart LM , Dale SK , Daffin GK , Kinial Patel K , Klein DJ , Mayer KH , et al. Pilot intervention for discrimination‐related coping among HIV‐positive black sexual minority men. Cultur Divers Ethnic Minor Psychol. 2018;24(4):541–51. 29902020 10.1037/cdp0000205 PMC6188818

